# Inducible Tertiary Lymphoid Structures: Promise and Challenges for Translating a New Class of Immunotherapy

**DOI:** 10.3389/fimmu.2021.675538

**Published:** 2021-05-14

**Authors:** Shota Aoyama, Ryosuke Nakagawa, James J. Mulé, Adam W. Mailloux

**Affiliations:** ^1^ Department of Surgery, Institute of Gastroenterology, Tokyo Women’s Medical University, Tokyo, Japan; ^2^ Immunology Program, Moffitt Cancer Center, Tampa, FL, United States; ^3^ Cutaneous Oncology Program, Moffitt Cancer Center, Tampa, FL, United States; ^4^ Department of Microbiology and Immunology, University of Iowa, Iowa City, IA, United States

**Keywords:** immunotherapy, tertiary lymphoid structure (TLS), cancer, bioengineering, biomaterials

## Abstract

Tertiary lymphoid structures (TLS) are ectopically formed aggregates of organized lymphocytes and antigen-presenting cells that occur in solid tissues as part of a chronic inflammation response. Sharing structural and functional characteristics with conventional secondary lymphoid organs (SLO) including discrete T cell zones, B cell zones, marginal zones with antigen presenting cells, reticular stromal networks, and high endothelial venues (HEV), TLS are prominent centers of antigen presentation and adaptive immune activation within the periphery. TLS share many signaling axes and leukocyte recruitment schemes with SLO regarding their formation and function. In cancer, their presence confers positive prognostic value across a wide spectrum of indications, spurring interest in their artificial induction as either a new form of immunotherapy, or as a means to augment other cell or immunotherapies. Here, we review approaches for inducible (iTLS) that utilize chemokines, inflammatory factors, or cellular analogues vital to TLS formation and that often mirror conventional SLO organogenesis. This review also addresses biomaterials that have been or might be suitable for iTLS, and discusses remaining challenges facing iTLS manufacturing approaches for clinical translation.

## Introduction

The presence of infiltrating immune cell populations is a prominent histological feature of most solid tumors that with some exceptions (1, 2), often confers positive prognostic significance across a wide spectrum of indications (3). This benefit is often contingent on the number and phenotypic makeup of the immune infiltrate, and on the ratios of beneficial effector cells to immune suppressive populations (3, 4). This may entail elevated numbers of activated CD8^+^ cytotoxic T cells (T_C_), type-I polarized CD4^+^ helper T (T_H_1) cells, and B cells, signifying an adaptive anti-tumor immune response (3, 5, 6). In a similar fashion, infiltrating antigen-presenting cells such as macrophage and dendritic cells (DC) confer positive prognostic value in many tumor types (7, 8), and in particular those antigen presenting cells with type I polarization attributes are especially equipped to support anti-tumor immunity (9, 10). It is an important understated fact that elements associated with antigen presentation and immune polarization are found inside solid tumors and confer prognostic benefit alongside effector lymphocyte populations. This infers that active antigen presentation, and the structural organization needed to support it, must occur at the tumor site; thus, an anti-tumor immune response is not limited to remote activation of effector lymphocytes in draining secondary lymphoid organs (SLO), but also occurs locally within and proximal to the tumor mass (4).

It is now understood that many tumors are associated with the presence of tertiary lymph node structures (TLS) (11). TLS consist of structural features analogous to conventional SLO, including discrete B cell zones, T cell zones, marginal zones with activated macrophage and DC, reticular fibroblast cell (RFC) networks (or RFC-like stromal networks), and vasculature permissive to immune cell extravasation (11–13). In mature TLS, this high level of organization can consist of networks of supportive infrastructure are compartmentalized just as they are in SLO, with activated mature DC supporting T_H_1 activation in T cell zones (14, 15), and follicular DC localizing to B cell zones in support of humoral immunity (16, 17). TLS form *de novo* in the microenvironment of solid tissues in response to protracted inflammatory stimuli, and may dissipate upon the resolution of inflammation (18). TLS can additionally foster tumor antigen presentation and T cell activation, including germinal centers (19, 20), B cell class switching (21), activated antigen presenting cells (22), and T cell clonal expansion (23, 24). In human cancers, TLS are associated with better disease outcomes across a broad spectrum of indications including ovarian (25, 26), metastatic melanoma (27, 28), breast (29, 30), colorectal (11, 31), and non-small cell lung cancers (7, 14), and can augment the efficacy of immunotherapies such as imune chackpoint inhibitors (28). In murine models, TLS can reduce orthotopic growth of colon carcinoma (32), melanoma (33), and fibrosarcoma (34). These associations and clear demonstrations of beneficial anti-tumor immunity by TLS embody a majority of scenarios that are overwhelmingly positive in nature and that provide a strong basis for pursuing the artificial induction of TLS as a therapeutic modality. However, there are reports in which TLS are associated with negative prognostication or disease progression. This is best exemplified in hepatocellular carcinoma (HCC) (35), and suggests that while TLS represent an integral part of the anti-tumor immune response, their function is likely influenced by a number of contextual signals, including those afforded by local stroma, secreted inflammatory factors, other resident immune populations, local vasculature, and epithelium (36). This may also indicate that different types of TLS exist that are susceptible to immune polarization or can even serve an immune suppressive role depending on and subsequent to microenvironmental context (37). This review will focus on approaches that can be taken to artificially induce TLS as a novel immunotherapy or as a means of augmenting immunotherapies. The prognostic value of TLS has been well reviewed (38, 39).

The clear benefit of TLS has prompted investigation into their potential therapeutic use, both as a standalone treatment or as an adjuvant to adoptive transfer-based cell therapies (40, 41). As such, artificial or inducible TLS (iTLS) hold great promise as a novel immunotherapy, but significant challenges must first be overcome that preclude their advent. These challenges range from knowledge gaps in basic TLS biology to complexities associated with clinical grade biomaterials and autologous cell processing. This review provides an overview of what strategies have been and might be employed to artificially induce TLS, how iTLS may be employed as a novel therapeutic, and what technical difficulties must be addressed prior to manufacturing iTLS at a clinical level.

## Lessons From SLO Organogenesis: Strategies for Therapeutic TLS Induction

TLS formation is a complex process incorporating many processes that overlap conceptually with conventional SLO organogenesis (42), although multiple contextual and spacial constraints add complexity to TLS formation. In addition, not all TLS develop to the same level of structural and functional maturity. The level of TLS organization, what ectopic factors contribute to their function and development and how these factors play into prognostication have been well reviewed (36). In this review, we focus on approaches to artificially induce TLS formation, which have thus far been guided by our understanding of shared pathways between SLO organogenesis and natural TLS formation. Any successfully implemented iTLS will likely be subject to the same functional and organizational variations as seen in natural TLS caused by diverse microenvironmental cues present in different organs and indications. Thus, any clincial translation of iTLS must expect disease-specific challenges and variations regarding efficacy.

SLO initiate during embryogenesis following expression of lymphotoxin alpha-1, beta-2 (LTα1β2) on specialized lymphoid tissue inducer cells (LTi) (43) that binds to lymphotoxin beta receptor (LTBR) expressed on lymphoid tissue organizer cells (LTo), an early mesenchymal-derived fibroblast (42). Engagement of LTBR induces the expression of numerous NF-κB target genes (44, 45) that orchestrate the recruitment of different immune cells. NF-κB signals *via* two separate pathways, canonical and non-canonical. Canonical signaling leads to the translocation of p50/RelA dimers to the nucleus, where they induce CCL4, CXCL2, and CCL2 among other gene targets, while non-canonical NF-κB signaling leads to the translocation of p52/RelB dimers to the nucleus inducing CXCL13, CCL19, and CCL21 (44). This results in the recruitment of early CD11c^+^ myeloid populations followed by mass immigration of B and T cells which segregate into discrete T cell zones and B cell follicles (46, 47). This influx of lymphocyte subsets coincides with the LTBR-dependent development of high endothelial venules (HEV), and is followed by the LTBR-dependent appearance of antigen-presenting cells such as follicular DC (FDC) (48–50). As the SLO develops, LTo cells differentiate into RFC through continued LTBR signaling (51). Importantly, SLO formation requires both NF-κB signaling pathways to properly develop, although the non-canonical pathway appears more indispensable (45). In adults, there is no clear evidence that LTi or LTo cells persist, and so for TLS formation it is less clear which cell types fill these roles. However, overexpression of LTα1β2 markedly increase TLS (2), whereas LTBR blockade prevents TLS in murine models (52); this suggests that any cell expressing LTα1β2 has the potential to function as a LTi analogue, and any LTBR^+^ stromal cell capable of chemokine production has the potential to function as a LTo analogue. Importantly, most mesenchymal-derived stroma throughout the body expresses LTBR (53), including RFC (54). Additionally, LTα1β2 is expressed on activated T cells, B cells, and dendritic cells (55), giving this signaling axis wide-reaching potential for TLS induction if the correct environmental inflammatory cues are met. Importantly, engagement of LTBR on many types of mesenchymal-derived stroma induces analogous expression of both canonical and non-canonical NF-κB target genes as compared to LTo, including the lymphoid tissue homeostatic cytokines CXCL13, CCL19 and CCL21 necessary for SLO development (1). In addition to LTα1β2, another LTBR ligand, homologous to lymphotoxin, exhibits inducible expression and competes with HSV glycoprotein D for binding to herpesvirus entry mediator, a receptor expressed on T lymphocytes (LIGHT) effectively elicits chemokine gene targets on LTBR^+^ stroma through NF-κB (56). LIGHT signaling can also lead to TLS formation, and while also binding to other receptors such as Herpesvirus entry mediator (HVEM), LIGHT acts analogous to LTα1β2 in its capacity to induce TLS formation (34, 57).

The role of chemokines in both SLO organogenesis and in TLS formation cannot be understated. For SLO, the narrow set of homeostatic chemokines required for organogenesis reflects the chemokine receptor patterns expressed by naïve and resting memory T cells, and coincides with the chemokine receptors expressed by DC and macrophage (58). Activated lymphocytes follow different trafficking patterns throughout the periphery owing to the downregulation of SLO-homing chemokine receptors and the up-regulation of alternative chemokine receptor sets that allow for emigration from SLO and infiltration into inflamed peripheral sites (59). It is thus unsurprising that gene signatures associated with TLS formation in tumors encompass not only SLO-associated homeostatic chemokines, but many other chemokines capable of recruiting lymphocytes in various stages of activation and effector function and that are associated with peripheral lymphocyte trafficking (13). A TLS gene signature which incorporates 12 chemokines (12CK-GES) that was associated with better patient survival independent of tumor staging, was first identified in patients with colorectal carcinoma (11) and was soon after used to predict the presence of TLS in wide range of tumor types including melanoma, lung, breast, and colorectal (13, 29). Importantly, nine of the chemokines identified in the 12CK-GES have reported up-regulation by LTBR signaling in mesenchymal-derived stroma through canonical or non-canonical NF-κB signaling, whereas the remaining three are hallmark products of tumor-associated macrophages (TAM), or type-II polarized macrophages (60–64) which themselves are recruited by multiple members of the 12CK-GES (**Table 1**). Insights from the 12CK-GES, and the parallels to SLO organogenesis can easily lead one to speculate that TLS form *via* a sequential or semi-sequential recruitment of immune subsets in response to chronic LTBR stimulation, and that any chemokines in the 12CK-GES not directly produced by LTBR^+^ stroma might be indirectly accounted for by subsequently recruited immune populations. In addition to TLS-associated chemokines, LTBR signaling also regulates the expression of a number of homeostatic cytokines and growth factors important to SLO organogenesis and to TLS formation, including IL-7, IL-15, and B cell activating factor (BAFF) (44, 72). However, to prove that any components of TLS organization form through sequential recruitment steps requires an experimental model of TLS formation in which temporal data can be acquired. When considering TLS induction as an anti-cancer therapeutic, such models may be necessary to deduce which components of TLS formation are required for anti-tumor activity, and which component might be expendable for anti-tumor effect.

**Table 1:** 12CK-GES and associated NF-κB signaling pathways.

Chemokine signature	LTBR target	NF-κB pathway	Reference	Recruitment potential	Cognate Receptor(s)
CCL2	Yes	Canonical	(44)	T, M, MDSC, TAM	CCR2, CCR3,
CCL4	Yes	Canonical	(44)	M, MDSC, TAM	CCR5
CXCL10	Yes	Canonical	(65)	T, NK, TAM	CXCR3
CXCL11	Yes	Canonical	(66)	T, NK, TAM	CXCR3,
CCL5	Yes	Non-canonical	(67, 68)	T, M	CCR1, CCR3, CCR5
CCL19	Yes	Non-canonical	(44)	T, DC	CCR7
CCL21	Yes	Non-canonical	(44)	T, DC	CCR7
CXCL13	Yes	Non-canonical	(44)	B	CXCR5
CCL3	Yes	Canonical	(69)	M, T, NK, DC	CCR1, CCR5
CCL8	No	Canonical	(69)	M, NK, T, B, DC	CCR2
CCL18	No	No	(70)	DC	CCR8
CXCL9	No	No	(71)	T, NK	CXCR3

Beneficial Cell Types: T, T cell; B, B cell; M, monocyte/macrophage; NK, Natural Killer Cell; DC, Dendritic Cells.

Suppressive Cell Types: MDSC, Myeloid-derived Suppressor Cell; TAM, Tumor-associated Macrophage.

**Figure 1:**
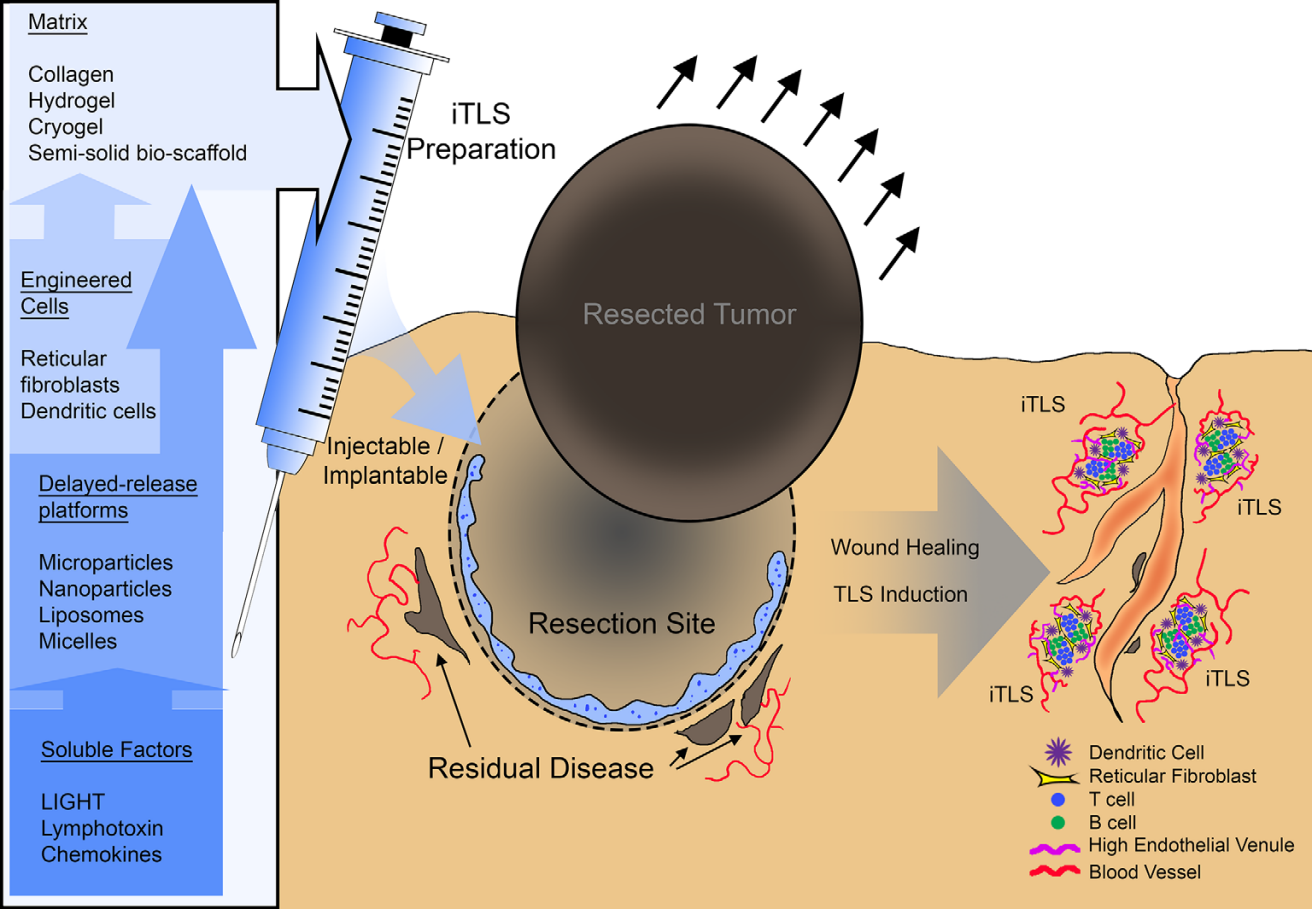
A summary schematic of potential therapeutic use of inducible tertiary lymphoid structures (iTLS). iTLS preparations may include cellular components such as dendritic cells, or reticular fibroblasts modified to engage the LTBR pathway, or co-delivered with soluble LTBR-ligands *via* a delayed-release platform such as liposomes, nanoparticles, or micelles. Injectable or implantable iTLS preparations could be administered at the site of tumor resection to induce TLS and subsequently control residual disease or counteract reoccurrence.

Strategies for iTLS can either utilize methods to initiate sustained LTBR signaling, thereby taking advantage of the same cascade of events that leads to naturally occurring TLS, by introducing cellular components engineered with constitutively active LTBR or with transgenic expression of LTBR gene targets, by some combination of the above approaches, or by complete TLS manufacture ex vivo prior to adoptive transfer/retransfer (**Figure 1**). iTLS methods that involve the introduction of isolated or cultivated cellular components have an additional appeal beyond the iTLS itself. The central role that antigen presenting cells play in TLS formation and function (73) and the effector cell-recruiting potential TLS create in the tumor microenvironment (57) make iTLS an ideal platform for the delivery of DC-based anti-tumor vaccines or as an adjuvant for chimeric antigen receptor-transduced T cell (CAR-T) or tumor-infiltrating lymphocyte (TIL) adoptive transfer therapies. Given the breadth of possible approaches, novel iTLS-based therapies can be designed with goals ranging from early interventional to multi-modal combination therapies that bridge cellular therapies, immunotherapies, and/or chemotherapeutic and radiation therapies.

Early demonstrations that the LTBR-chemokine axis can be utilized for iTLS occurred in transgenic model systems in which mice overexpressing chemokines or LTα developed lymph node-like structures in certain tissues (74). In mice expressing LTα under the rat insulin promoter, a promotor with transgene expression limited to pancreatic beta cells and proximal tubule of the kidney, TLS formation was observed in both the pancreas and kidney, especially in proximity to vasculature where the formation of HEV was evident (74). Under the same promoter, transgenic CCL21 induced HEV-containing TLS in the pancreas as well (75, 76), but using a promoter with skin-specific CCL21 expression did not result in TLS; these data suggest that additional cues or microenvironmental constraints are needed for TLS beyond CCL21 expression alone (75). Other examples include LTBR ligand targeting strategies, such as delivery of recombinant LIGHT tagged to vascular-targeting peptide. This chimeric compound induced TLS in pancreatic neuroendocrine tumors and in glioblastoma in areas surrounding dense vasculature that contain discrete B cell zones, T cell zones, macrophage, HEV, and DC (32, 77). Delivery of LTBR ligands to induce TLS is also possible *via* adoptive cell transfer of transgenic cells. DC transduced with the type-I polarizing transcription factor T-bet induced the expression of LIGHT and LTα, and subsequently CCL21, when injected into murine colon adenocarcinoma, slowing tumor growth (73, 78). Lastly, delivery of LTBR^+^ stroma demonstrates functional TLS formation when injected subcutaneously juxtaposed to established MC-38 murine colon carcinoma tumors, slowing tumor growth and actively priming T cell response (79). Despite the precedent that TLS can be induced by taking advantage of the LTBR-chemokine axis, the greatest benefit from iTLS will likely result from injectable or implantable preparations that do not require additional microenvironmental cues from the recipient host. This way, they may be applied to a wider range of tissues and organs as part of a microenvironment reprogramming strategy. Discussed below are biocompatible matrixes and micro/nanoparticles that may be suitable as scaffolding material for iTLS or for the sustained release of biologics aimed at TLS induction respectively.

## Biomaterials With Proposed Suitability for iTLS

A variety of three-dimensional materials that are permissible to cellular infiltration and that may allow for cell-scaffold interactions have been used for tissue engineering, regenerative medicine, and ex vivo scientific investigation (80). Some bio-scaffolds derived from animal or cell-based products such as Matrigel^®^ (Corning Life Sciences) have a decades-long precedent. These products isolate entire acid-soluble constituents of tumor cell line-derived extracellular matrix, and thus represent a more physiologically complete microenvironment than synthetic scaffolds. However, such products are not immunologically inert, and contain growth factors and biologically active components that have well described angiogenic, adipogenic, and inflammatory properties (81). This is even true of “growth factor reduced” product versions (albeit improved) (81, 82). As such, use of such cell or animal-derived matrixes often invites scientific scrutiny (83), and thus more advanced synthetic alternatives are typically sought for bioengineering endeavors which contain less lot-to-lot variation, display a more immunologically inert background, and lack any confounding variables caused by biologically active carryover components (81).

### Collagen Matrixes

Collagens constitute the major framework of the extracellular matrix (ECM) with distinct primary, secondary, tertiary, and quaternary structures that create a range of ECM scaffold superstructures in different tissues and organs from rope-like fibrils to web-like networks, anchoring structures, and can even complex with transmembrane collagens (84). In vertebrates, at least 29 types of collagen are coded by at least 45 separate genes (85). Given the complexity of collagen, tissues from animals or human-sourced raw materials are often used to make collagen-based scaffolding materials (86). Recombinant sources of collagen monomers or peptides are commercially available (87) but small yields and the lack of the tertiary and quaternary structural complexity that would be afforded by multi-collagen type complexes limit their use as a scaffold for bioengineering (86). Most collagen products are manufactured in one of two ways: from decellularization of existing ECM, resulting in an intact ECM superstructure (88), or through the breakdown, solubilization, extraction, and reformulation of collagen, often with the addition of crosslinking agents such as glycosaminoglycans (89, 90), elastins (91, 92), or chitosans (93, 94). The precise methodologies used to manufacture commercial collagen matrix products should be carefully considered prior to implementation in iTLS approaches since most collagen-based matrixes are derived from animal or human tissues (86). While more purified than bulk ECM products like Matrigel^®^, biologically active impurities may still carry over from raw materials (95). A major strength of collagen-based matrixes is their versatility in available formats, including sheets, sponges, disks, granulated tablets, or even nano-scale spheres (96).

One of the first successful demonstrations of iTLS using implantable artificial matrix was performed by Suematsu and Wantanabe using a collagen sponge biomatrix impregnated with a thymus-derived stromal cell line modified to constitutively express LTα. Following implantation, functionality of iTLS was demonstrated by vaccination with 4-hydroxy3-nitrophenylacetyl-ovalbumin (NP-OVA). Three weeks after subcutaneous implantation, discrete B cell and T cell zones formed in the stromal cell-impregnated collagen sponge with interacting DC, as well as HEV-like structures. Recipient mice that were vaccinated with NP-OVA produced anti-NP-OVA IgG-producing B cells within iTLS. This effect was bolstered if NP-OVA-pulsed DC were included in preparations. Furthermore, these NP-OVA-primed iTLS could be resected after their formation, and transplanted to syngeneic recipient mice wherein they could mount effective secondary response to NP-OVA (97). These iTLS were later shown to be able to produce a potent secondary immune response when transplanted into severe combined immunodeficiency (SCID) recipient mice, repopulating SLO and bone marrow with anti-NP-OVA IgG-producing B cells (98). An alternative approach using chemokine and VCAM-1-loaded collagen matrix instead of stromal cells soon followed, recapitulating the successes of stromal-cell line-loaded collagen sponges. These chemokine-induced TLS incorporated B cell zones, T cell zones, and supporting DC and were able to prime a similar anti-NP-OVA response. HEV were not interrogated in these iTLS (99).

### Hydrogels

Hydrogel refers to a large class of biomaterials that are made of three-dimensional crosslinked polymers with a large capacity for water uptake and retention that are prepared in aqueous solution (100). Depending on the types of polymers and crosslinking reagents used, a wide variety of hydrogels can be prepared that have different properties regarding biologic interactivity or inertness, physical rigidity or elasticity, temperature sensitivity, pH sensitivity, shape memory properties, or capacity to carry and deliver soluble drugs or cellular payloads (101–103). Hydrogels can be classified according to physical structure, charge, size or chemical properties (102). Of relevance to iTLS efforts, most hydrogel preparations can be injectable or transplantable depending on the timing and chemistries of the crosslinking steps, and can be formulated in large injection/implantation volumes or in micro or even nanoscale particle preparations (104). Because of their large capacity to hold aqueous solution, many hydrogel preparation methodologies are widely compatible with cell culture conditions so long as the chemistries required for crosslinking do not stray from physiologic pH, temperature, salinity, and other cell culture ranges (105–107). For this reason, crosslinking chemistries that utilize mild temperature or pH changes such as warming from 4°C to 37°C or changing from a slightly acidic solution to slightly basic are ideal for injectable preparations, as such hydrogels would crosslink *after* injection into living recipients (105, 107). When used as an *in vitro* model for iTLS, hydrogel preparations with BAFF-producing stromal cells and IL-4 support the compartmentalization, expansion, and class-switching of primary B cells (108, 109). When used as a model of cell therapy delivery, hydrogels have been successfully deployed to carry CAR-T cells in conjunction with delivering stimulator of IFN genes (STING) agonist cyclic di-GMP. This preparation was then placed in resection sites of murine pancreatic tumors, or alongside pancreatic tumors mimicking non-resectable masses. These implants produced a significant anti-tumor effect compared to intravenously delivered CAR-T, activated host DC, and induced significant infiltration of immune cells at the implantation sites (110).

Cryogels, a subset of hydrogels prepared at sub 0°C, may be of particular interest to iTLS efforts. Their preparation creates a larger pore size than typical hydrogels, which are typically measured in the nanometer range. Such a small pore size requires hydrogel breakdown or active turnover by infiltrating populations to emigrate or infiltrate transferred materials, significantly limiting cellular involvement (111, 112). Cryogels are formed when polymer and cross linkers are displaced by ice crystal formation, causing concentration spikes in localized spaces in between ice crystals. When the cryogels are subsequently thawed, the space occupied by the ice crystals leaves a porous network measured on a micron scale or larger (113, 114). Thus, cryogels provide a more cell-invasive alternative to conventional cryogels, although, their freeze/thaw preparation method requires any would-be cellular components to be loaded after formation and precludes any formation post injection, somewhat limiting their use to implantation. Cryogels have been be used to deliver chemotherapies and cancer vaccines (115), and when incorporated with DC activating components and tumor antigen vaccination strategies, induces the recruitment of DC and lymphocytes (113, 116, 117), although the organization of these infiltrates was not investigated.

### Other Solid or Semi-Solid Bio-Scaffolds

A wide array of scaffolding material distinct from hydrogels or tissue-derived collagen matrixes have been derived for use in tissue repair, wound-healing, or other bioengineering endeavors (118). Among these are matrixes made from mesoporous silica rods, which have been used to boost the immunogenicity of tumor antigen peptide-based vaccine approaches in mice bearing B16F10 melanoma or CT26 colon carcinoma tumors. In these models, tumor associated antigen pools were loaded into mesoporous silica rod matrixes and injected subcutaneously. While the organization of infiltrates was not analyzed histologically, vaccination using this matrix approach greatly enhanced lymphocyte infiltration, and activation, as well as mediated anti-tumor effects on lung nodule growth (119). While not evidence of iTLS, this study does provide precedence for silica as a biomaterial supportive of lymphocyte recruitment, DC activation, and antigen priming. Another bio-matrix that may be permissive to iTLS are polyamide fiber preparations. In what represents one of the earliest attempt to manufacture iTLS entirely in situ, non-woven sheets of polyamide fibers were loaded with antigen-primed primed DC (in this case, cytomegalovirus lysate) and sealed inside a closed and chambered bioreactor system in which lymphocytes isolated from peripheral blood mononuclear cells (PBMC) were continuously circulated. After two weeks, this bioreactor approach resulted in TLS-like structures with discrete B cell and T cell clusters around DC inside the polyamide fiber sheets. Cytokine production suggests that these lymphocytes were activated by the primed DC (120).

#### Micro and Nanoparticles in iTLS

While bio-scaffolds can, in some instances, impart delayed release of soluble factors, another class of biomaterials has been refined over time with delayed-release of soluble factors as one of several defining characteristics. Microparticles or nanoparticles represent entire fields of materials science in their own right (121). Here, microparticles and nanoparticles are proposed as cooperative biomaterial elements that can be used as an incorporated element within bio-scaffolding to mediate controlled release of chemokines, LTBR ligands, or other TLS-inducing factors. Of these, liposomes represent the most studied and characterized class of micro or nanoscale biomaterials that can serve as carriers for a wide range of compounds. Liposomes form when lipid components assemble into spherical bilayers leaving an aqueous compartment that carry a water-soluble payload (122). The physical properties of liposomes can be easily controlled by altering the types of lipids used for incorporation (123–126), and their size can be controlled by deploying different production methods such as sonication, which delivers liposomes in the 20-40 nm range (127), microfluidic mixing in the 20-80 nm range (128, 129), high pressure homogenization in the 20-140 nm range (130), flow focusing in the 50-150 nm range (131), and extrusion in the 70-415 nm range (132). Many liposomes are already incorporated as part of FDA-approved drugs (133, 134) giving precedent to their clinical translatability and patient safety, and there are now multitudes of methodologies that can deliver on a wide range of specifications, cost, uniformity, and bulk manufacturing requirements (135).

Unmodified liposomes were first described 55 years ago (136), and while successful, have limitations due to their unprotected outer lipid surface, making them subject to fusion with other liposomes as natural result of surface tension reduction (137, 138). Unmodified liposomes are also susceptible to opsonization of serum protein following injection which can lead to phagocytic uptake, or clearance in the liver (139, 140). Any such alteration manipulates drug release kinetics or limits payload delivery to intended targets. A second generation of liposomes was created by modifying liposomal surfaces with integrated polymers, providing structural stabilization and interfering with serum protein binding (139, 141), the most successful of which has been polyethylene glycol (PEG) (142, 143). Additional modification strategies have been employed to liposomes within the last 20 years that not only aim to stabilize liposomal formulations, but to also impart drug target selectivity or more precisely control drug release. Examples of such modifications include liposomes with surface-attached bioactive ligands, such as aptamers, peptides, or most commonly immunoglobulin or immunoglobulin fragments (144). By incorporating moieties with known affinities for antigens expressed on target tissues (or tumors), liposomes can then specifically interact with intended targets. Such incorporations can be accomplished by including recombinant protein products in the initial lipid formulations that either naturally contain hydrophobic regions or that are themselves modified to contain hydrophobic regions, or by covalent binding to hydrophilic regions of incorporated lipids (145). Of particular relevance to iTLS, liposomes can offer extended release kinetics of their payloads, or even be designed for content release in response to an external trigger, such as temperature (146), magnetic fields (147), or light (148, 149). In addition, liposomes are compatible with other bio-scaffolding materials such as hydrogels, cryogels, or polymeric matrixes (150), and have a demonstrated ability for delayed release of chemokines such as CXCL13 (151).

Other nanoscale particles capable of delivering or releasing inflammatory mediators include micelles which are created by self-assembling amphiphilic polymers (152, 153). Micelles have proven capacity to deliver cytokines (154), antigens (155), and interfering RNAs (156, 157), and thus represent a plausible alternative to liposomes to deliver factors for iTLS. In addition, nanoparticles made from aliphatic polyesters (PLGA) are also an attractive option to incorporate delayed-release of soluble factors, and are already FDA-approved in many clinical contexts (158–160). PLGA nanoparticles are biodegradable, and like micelles, are capable of tumor antigen delivery (161, 162) and immunotherapeutic biologics (163, 164). In what is perhaps the most robust example of iTLS generated in animal models, Kobayashi and Watanabe combined microscale gelatin-based hydrogels loaded with LTα1β2, CCL19, CCL21, CXCL12, CXCL13, and soluble RANKL inside a macroscale implantable collagen sponge. This preparation was then implanted onto the kidney capsule of recipient mice, and after three weeks produced mature iTLS with discrete T cell zones, B cell zones, RFC networks, FDC in what appears to be a marginal zone, HEV, and lymphatic vasculature. These iTLS were also able to prime primary and secondary IgG responses to NP-OVA (165). Another example of combined biomaterial approaches utilized lipid-coated silica microspheres harboring IL-15/IL-15Ra fusion proteins and anti-CD3, anti-CD18, and anti-CD137 antibodies to act as artificial antigen-presenting cells inserted into alginate hydrogels loaded with NKG2D-CAR-expressing murine T cells. This construct, when implanted next to partially resected established subcutaneous 4T1 breast cancer tumor-bearing mice, elicited significant anti-tumor reactivity and slowed tumor growth (166). While not necessarily iTLS, this study further establishes precedent that combination biomaterials can deliver and expand effector lymphocytes.

### Challenges Awaiting iTLS for Clinical Translation

#### Avoiding Foreign Body Response for Incorporated Biomaterials

One general drawback to the use of biomaterials is the induction of a foreign body response (FBR), an acute inflammatory reaction against the material itself (167). FBR can result in a wide range of unintended consequences including but not limited to vascularization, fibrotic encapsulation, and infiltration of innate immune cells (168, 169). Neutrophils and macrophages are among the earliest effector cells responding to the FBR and can destroy implanted biomaterials through the release of cytotoxic granules, reactive oxygen species, proteolytic enzymes, and phagocytosis (170–173). Of particular importance to iTLS, recruited and activated macrophages and neutrophils produce high levels of chemokines associated with FBR such as CXCL8, CCL2, CCL4 (174, 175). While CCL2 and CCL4 are part of the 12CK-GES associated with TLS presence in human tumors, CXCL8 is not (11), and none of these chemokines encompass the SLO homeostatic chemokines CXCL13, CCL19 and CCL21 previously used for iTLS, as discussed above. In addition, physical macrophage adherence to many biomaterials polarizes them to a M2 phenotype, which may be detrimental to iTLS formation for anti-tumor immunity (176). To avoid a FBR, it is advantageous to select biomaterials with low antigenicity that have little or no carryover of soluble factors from animal sources (167). In addition, biomaterial topography has also been identified as a contributing factor to FBR, and thus biomaterial size, shape, and texture can be modified to minimize FBR (177, 178). However, any such measure would need to be weighed against the need to recruit cellular infiltrate as part of the iTLS.

#### Generating cGMP Materials for iTLS Manufacture

As ever more complex components and methodologies are used to innovate iTLS as a potential therapy, so too do the challenges associated with translating such approaches to the clinic. To fully qualify for the Food and Drug Administration’s (FDA) approval, components used to make any would-be iTLS therapy need to graduate to clinical-grade materials by the time pivotal trials are conducted, meaning the components themselves must be manufactured under cGMP conditions (179). Not only does this add difficulty to the process, but in almost every scenario, results in elevated manufacturing cost (180, 181). Cell therapies utilizing one cell type with one gene modification can easily exceed $400,000 per dose due in no small part to the elevated cost of manufacturing cell therapies at a cGMP level (182). Given the potentially multimodal processes involved in iTLS development, this new class of immunotherapy may incur clinical-grade manufacturing costs reflective of cell therapies, biomaterials, and biologics combined. In addition to cost, there are also regulatory challenges. Biomaterial products which contain no cellular or bioactive components, such as an inert scaffolding material, might be considered from a regulatory perspective as a “medical device” depending on their mode of action, but should such material be combined with cellular components or biologics, it will most certainly be considered a drug (183). Careful consideration will then need to be taken when defining what components are drug product versus drug substance. Conventionally, the drug substance is whatever components entail the “active ingredient(s),” whereas the drug product is the entirety of the components and compositions used in the manufacturing process. These definitions are critical to the regulatory success of new investigational drugs, but may be less clear for iTLS, which may combine novel biomaterials with varying amounts of bioactivity (184), with active biologics and cell therapies which may contain genetic modifications. Similar to the advent of cell therapies over the past few decades, the clinical translation of bioengineered constructs such as iTLS may be codependent on the FDA’s creation of a new guidelines that are developed in concert with the scientific field (185).

## Concluding Remarks

The progress toward utilizing TLS as a therapeutic intervention has made great strides over the last few decades and has come to incorporate many new and technologies, particularly in the biomaterials space. iTLS have to potential to become an entire new class of immunotherapy combining elements of biologic compounds, cellular therapeutics, and biofabrication techniques. However, significant challenges and unanswered questions remain. These include identifying the optimal bio-scaffold/nanomaterial combinations for sustained release of TLS-inducing soluble factors and identifying the minimal combination of chemokines, LTBR ligands, and other soluble factors required for robust iTLS formation. Another prominent point of consideration is to further evaluate if stromal and/or DC components are needed for iTLS approaches. Their involvement has been critical to early iTLS successes, but recent advances demonstrate iTLS can be achieved using cell-free constructs (165). This would have obvious benefits when translating to clinical-grade manufacturing processes, and allow for less costly clinical translation.

## Author Contributions

SA and RN performed literature searches, outlined sections, and reviewed and edited the manuscript, JJM reviewed and edited the manuscript, and provided funding, AWM wrote the manuscript, created the figure and table, reviewed and edited the manuscript, and provided funding. All authors contributed to the article and approved the submitted version.

## Funding

This work was funded by the NCI-NIH (1R01 CA148995, 1R01 CA184845, P30 CA076292, P50 CA168536, and 5R21CA214285), Cindy and Jon Gruden Fund, Chris Sullivan Fund, V Foundation, and the Dr. Miriam and Sheldon G. Adelson Medical Research Foundation.

## Conflict of Interest

The authors declare that the research was conducted in the absence of any commercial or financial relationships that could be construed as a potential conflict of interest.
